# Erythropoietin (EPO) ameliorates obesity and glucose homeostasis by promoting thermogenesis and endocrine function of classical brown adipose tissue (BAT) in diet-induced obese mice

**DOI:** 10.1371/journal.pone.0173661

**Published:** 2017-03-13

**Authors:** Kazuki Kodo, Satoru Sugimoto, Hisakazu Nakajima, Jun Mori, Ikuyo Itoh, Shota Fukuhara, Keiichi Shigehara, Taichiro Nishikawa, Kitaro Kosaka, Hajime Hosoi

**Affiliations:** 1 Department of Pediatrics, Graduate School of Medical Science, Kyoto Prefectural University of Medicine, Kyoto City, Japan; 2 Department of Pediatrics, Ayabe Municipal Hospital, Ayabe City, Japan; 3 Department of Molecular Gastroenterology and Hepatology, Graduate School of Medical Science, Kyoto Prefectural University of Medicine, Kyoto City, Japan; East Tennessee State University, UNITED STATES

## Abstract

Erythropoietin (EPO), clinically used as a hematopoietic drug, has received much attention due to its nonhematopoietic effects. EPO reportedly has beneficial effects on obesity and diabetes mellitus. We investigated whether interscapular brown adipose tissue (iBAT: main part of classical BAT) could play a role in EPO’s anti-obesity and anti-diabetic effects in diet-induced obese mice. Four-week-old male C57BL/6J mice were fed a high-fat diet (HFD-Con), and half were additionally given an intraperitoneal injection of recombinant human EPO (200 IU/kg) (HFD-EPO) thrice a week for four weeks. At 8 weeks, EPO-injected mice showed significantly reduced body weight with reduced epididymal and subcutaneous white fat mass and unchanged caloric intake and locomotor activity. HOMA-IR (insulin resistance index) and glucose levels during intraperitoneal glucose tolerance test (IPGTT) were significantly lower in HFD-EPO mice than in HFD-Con mice. EPO-injected mice also showed increased oxygen consumption, indicative of metabolic rate, and skin temperature around iBAT tissue masses. EPO significantly upregulated the PRD1-BF1-RIZ1 homologous domain containing 16 (PRDM16), a transcriptional factor with a crucial role in brown adipocyte differentiation. EPO significantly increased phosphorylated signal transducer and activator of transcription 3 (STAT3), which is downstream of erythropoietin receptor (EpoR) and known to stabilize PRDM16. EPO’s suppression of myocyte enhancer factor 2c (Mef2c) and microRNA-133a (miR-133a) via β3-adrenergic receptor caused PRDM16 upregulation. EPO-mediated enhancement of EpoR/STAT3 and β-adrenergic receptor/Mef2c/miR-133 pathways dramatically increases total uncoupling protein 1 (UCP1), an essential enzyme for BAT thermogenesis. Furthermore, EPO activated BAT’s endocrine functions. EPO facilitated fibroblast growth factor 21 (FGF21) production and excretion in iBAT, associated with reduction of liver gluconeogenesis-related genes. Thus, EPO’s improvement of obesity and glucose homeostasis can be attributed to increased iBAT thermogenic capacity and activation of BAT’s endocrine functions.

## Introduction

Obesity and its comorbid diseases, including diabetes, cardiovascular disease, stroke, and some cancers, have increased dramatically and are now a worldwide health problem. Erythropoietin (EPO), a kidney-derived peptide hormone necessary to produce red blood cells in bone marrow, is prescribed for anemia patients who suffers from renal anemia and anemia of prematurity [1]. Recently, much attention has been paid to EPO because of its nonerythroid effects, including regulation of fat, glucose, and energy metabolism [2–14]. However, the mechanisms behind these effects remain unclear. Previous studies that investigated the mechanism of EPO’s anti-obesity and anti-diabetes effect have focused mainly on white adipose tissue, muscle and liver [7–16]. Our study investigated the mechanism underlying EPO’s anti-obesity and anti-diabetic effects on classical brown adipose tissue (BAT).

BAT increases energy expenditure by inducing thermogenesis in mammals, and has received much attention as a target in combating obesity and diabetes [17–20]. Thermogenic adipocytes are divided into two categories: classical brown adipocyte and beige (also referred as brite) adipocytes, each with distinct developmental and anatomical features [21,22]. Recently, BAT’s function as an endocrine organ in addition to its thermogenic function has garnered attention. BAT releases fibroblast growth factor 21 (FGF21), a peptide hormone that can alleviate obesity and diabetes in animal experiments [23–25], and which increases insulin sensitivity and reduces gluconeogenesis in the liver [26–30].

This study aimed to discover whether EPO acts on classical BAT to exert anti-obesity and anti–diabetic effect in mice fed a high-fat diet. We demonstrate that EPO increased the mass of interscapular BAT (iBAT, which is the main part of classical BAT) and thermogenesis by enhancing the brown adipocyte differentiation pathway. In addition, we demonstrate that EPO facilitated secretion of FGF21 in BAT, which contributes to alleviating glucose homeostasis. These findings support EPO’s potential as a therapeutic agent for obesity and diabetes.

## Materials and methods

### Animals and experimental procedures

Four-week-old male C57BL/6J mice were purchased from CLEA Japan (Tokyo, Japan) and housed in a temperature-controlled room at 23°C on a 12 h light/dark, with free access to food and water. Mice were fed normal chow (NC) (CLEA Rodent Diet CE-2: 12% of calories from fat, 59.1% of calories from carbohydrates, 28.8% of calories from protein; CLEA Japan) or a high-fat diet (HFD) (Clea High Fat Diet 32: 56. 7% of calories from fat, 23.1% of calories from carbohydrates, 20% of calories from protein; CLEA Japan) for 4 weeks. HFD-fed mice were randomly assigned to groups that were injected intraperitoneally (i.p.) either with 200 IU/kg of recombinant human EPO (Epoetin Alfa BS injection, JCR Pharmaceuticals Co., Ltd., Ashiya, Japan) (HFD-EPO group) or with saline (HFD-Con group) three times a week for four weeks. Mice fed the NC diet were also randomly assigned to receive either EPO (NC-EPO group) or saline (NC-Con group) injections on the same schedule. Food intake and body weight gain were monitored once a week. Blood was drawn from the tail into a heparinized capillary tube (ERMA Inc., Tokyo, Japan) once a week. Hematocrit value (Ht) was determined by centrifuging the heparinized blood. At 8 weeks, mice were fasted overnight and anesthetized and euthanized using sodium pentobarbital, 50 mg/kg, i.p., and blood was obtained by cardiopuncture. Plasma was separated by centrifugation at 4°C and stored at -80°C prior to analysis. Interscapular brown adipose tissue (iBAT), subcutaneous white adipose tissue (sWAT), epididymal white adipose tissue (eWAT), and liver were immediately dissected and weighed, then frozen in liquid nitrogen and stored at -80°C prior to further analysis. All animal experiments and care procedures were approved by the Animal Care and Use Committee of Kyoto Prefectural University of Medicine (Approval Number M25-76).

### Plasma parameters

Blood glucose was determined using a compact glucose analyzer (Antsense II; Horiba, Kyoto, Japan). Plasma insulin levels were measured with an ELISA kit (Morinaga Institute of Biological Science, Kanagawa, Japan, Cat. No. M1104). Plasma triglyceride (TG) and total cholesterol (T-Cho) levels were measured using reagents from Wako (Osaka, Japan). Hematocrit was measured manually every week after treatments were administered. Plasma FGF21 levels were measured with an ELISA kit (Mouse/Rat FGF-21 Quantikine ELISA Kit; R&D Systems, Minneapolis, MN, Cat. No. MF2100). All assays were performed according to the manufacturer’s instructions.

### Glucose tolerance test

Subsequent to EPO or saline control treatment over four weeks, mice were fasted overnight and injected intraperitoneally with glucose (1.0 g/kg body weight). Levels of blood glucose and insulin aspirated from the tail vein were monitored at 0, 30, 60, and 120 min after this glucose injection.

### Oxygen consumption

Oxygen consumption (VO_2_) was analyzed by an O_2_/CO_2_ metabolism-measuring system (model MK-5000, Muromachi-Kikai, Tokyo, Japan), which consists of two independent 560 ml chambers (for measuring two animals simultaneously), a suction pump, and a computer for data analysis. After four weeks’ treatment, mice were placed in the chambers at 23°C and allowed to acclimate to the environment for more than two hours. The pump draws air from one of the chambers for one minute at rate of 650 ml/min to measure O_2_ concentration every three minutes. VO_2_ was calculated as [O_a_-O_c_] v m^−1^ t^−1^, where O_a_ is the atmospheric oxygen concentration (%) that flows into the chamber, O_c_ is the oxygen concentration in the chamber (%), v is the flow rate (650 ml/min), m is the mass of the mouse in kg, and t is the time in hours [31].

### Interscapular temperature

Mice were fasted for 6 hours and then anesthetized using 30 mg/kg sodium pentobarbital, administered i.p. Skin temperature surrounding iBAT was recorded with a thermal imaging camera (FLIR i3, FLIR Systems, Tokyo, Japan) and analyzed with FLIR QuickReport software [31].

### Locomotor activity

Locomotor activity of each mouse was determined using a Supermex (Muromachi Kikai, Tokyo, Japan), as previously described [32], for 24 hours after four weeks’ experimentation. In brief, movements of each mouse were determined by detecting movement using infrared radiation. Activity was measured in units where a single count consisted of an animal’s movement from one section of the measurement area, which was optically separated by multiple lenses, to a neighboring section.

### Histology

Subcutaneous white adipose tissue (sWAT), epididymal white adipose tissue (eWAT) and iBAT were fixed in 10% buffered formalin. Sections (5 μm) were stained with hematoxylin and eosin (H&E). Slides were examined and photomicrographs were taken using an All-In-One Fluorescence Microscope BZ-X710 (Keyence, Osaka, Japan) at 40× magnification. Mean cell size and cell distribution of WAT was determined from 1600 adipocytes of each mouse by using BZ Analyzer software (Keyence). The number of nuclei in brown adipocytes over a randomly chosen area (320 μm × 270 μm) from each mouse was counted by the software in order to evaluate lipid accumulation in brown adipocytes.

### mRNA and microRNA quantifications by Quantitative real-time PCR

To analyze mRNA expression, total RNA was isolated from iBAT, sWAT, eWAT, and liver by a NucleoSpin RNA II kit (Macherey-Nagel, Düren, Germany, Cat. No. 740955.50). Template cDNA was synthesized from 500 ng total RNA with random hexamer primers as the template for each reaction in a ReverTra Ace qPCR RT Master Mix (Toyobo, Osaka, Japan, Cat. No. FSQ201). Quantitative real-time PCR (qRT-PCR) was performed using SYBR Premix Ex Taq II (Tli RNaseH Plus) (Takara, Shiga, Japan, Cat. No. RR820A) with 10 μM of each primer in an AB 7500 Real-Time PCR System (Applied Biosystems, Tokyo, Japan). Amplification was performed using the following protocol: initial activation step for 30 s at 95°C, followed by 40 cycles of 3 s at 95°C and 30 sec at 60°C. Oligonucleotide primer sequences are described in S1 Table. β-actin was selected as an internal standard. According to the protocol of TaqMan MicroRNA Assays (Applied Biosystems, Cat. No. 4427975), the expression of microRNA-133a (miR-133a) levels in iBAT were analyzed. Small RNA was extracted from iBAT using a NucleoSpin miRNA kit (Macherey-Nagel, Cat. No. 740971.50) and reversely transcribed into cDNA by a Taqman MicroRNA Reverse Transcription Kit (Applied Biosystems). Then, qRT-PCR of the transcribed cDNA was performed using a Taqman Universal PCR Master Mix on an AB 7500 Real-Time PCR System (Applied Biosystems, Cat. No. 4366596). Amplification was performed using the following protocol: initial activation step for 10 min at 95°C, followed by 40 cycles of 15 s at 95°C and 60 s at 60°C. Small nucleotide oligonucleotide 202 (sno202) was used as a housekeeping gene.

### Western blot analysis

Protein of iBAT was extracted by a radioimmunoprecipitation assay (RIPA) lysis buffer (Nacalai Tesque, Kyoto, Japan, Cat. No. 08714.04) or a NucleoSpin miRNA kit designed for the simultaneous isolation of small RNA and protein. (Macherey-Nagel, Düren, Germany, Cat. No. 740971.50). Protein concentrations were determined with a Protein Quantification Assay kit (Macherey-Nagel, Cat. No. 740967.50). Tissue proteins were resolved on 7.5%, 10%, or 12% polyacrylamide gels in the presence of sodium dodecyl sulfate, transferred electrophoretically to polyvinylidene difluoride membranes, and blocked by Blocking One (Nacalai Tesque, Cat. No. 03953.95). The primary and secondary antibodies were diluted with Can-Get Signal (Toyobo, Osaka, Japan, Cat. No. NKB-101). The membrane was washed and incubated with primary antibodies against erythropoietin receptor (EpoR) (1:3,000) (sc-697, RRID: AB 631468, Santa Cruz Biotechnology Inc., Dallas, TX, USA), phospholyrated-erythropoietin receptor (*p*EpoR) (1:3,000) (sc-20236-R, RRID: AB 2098548, Santa Cruz Biotechnology Inc.), signal transducer and activator of transcription 3 (STAT3) (1:3000) (#9132, RRID: AB 331588, Cell Signaling Technology, Tokyo, Japan), phospholyrated-signal transducer and activator of transcription 3 (*p*STAT3) (1:3000) (#9145, RRID: AB 2491009, Cell Signaling Technology), β3-adrenergic receptor (1:10,000) (β3ADR) (ab94506, RRID: AB 10863818, Abcam, Tokyo, Japan), peroxisome proliferator-activated receptor-α (PPARα) (1:10,000) (ab8934, RRID: AB 306869, Abcam), peroxisome proliferator-activated receptor-γ (PPARγ)(1:10,000) (ab27649, RRID: AB 777390, Abcam), PRD1-BF1-RIZ1 homologous domain containing 16 (PRDM16) (1:12,500) (ab106410, RRID: AB 10866455, Abcam), uncoupling protein 1 (UCP1) (1:15,000) (ab10983, RRID: AB 2241462, Abcam), proliferator-activated receptor gamma coactivator 1α (PGC1α) (1:10,000) (ab54481, RRID: AB 881987, Abcam), fibroblast growth factor-21 (FGF21) (1:5,000) (sc-292879, Santa Cruz Biotechnology Inc.), and β-actin (1:15,000) (#3700, RRID: AB 2242334, Cell Signaling Technology). The secondary antibody consisted of a 1:15,000 dilution of HRP-conjugated donkey anti-rabbit IgG (for EpoR, *p*EpoR, STAT3, *p*STAT3, β3ADR, PPARα, PPARγ, PRDM16, UCP1, PGC1α, and FGF21) (GE Healthcare, Tokyo, Japan, Cat. No. NA934V) or HRP-conjugated sheep anti-mouse IgG (for β-actin) (GE Healthcare, Cat. No. NA931). The immunocomplexes were detected using an enhanced HRP-luminol chemiluminescence system (ECL prime) (GE Healthcare, Cat. No. RPN2232), and signals on the immunoblot image were quantified using ImageQuant LAS 500 Gel Documentation System (GE Healthcare). To compare the results for protein expression, we assigned a value of 1 to expression in iBAT from NC-Con mice.

### Statistical analysis

All data are expressed as mean ± SEM. A Student's *t*-test was used to compare the means of two groups. Repeated measurements of analysis of variance (ANOVA) with Tukey-Kramer *post hoc* tests were performed for multiple comparisons. Differences were regarded as statistically significant at P values less than 0.05.

## Results

### EPO reduced body weight gain accompanied with reduction of white adipose tissue (WAT), ameliorated insulin resistance, and glucose intolerance in high-fat diet-induced obese mice

**Fig 1:**
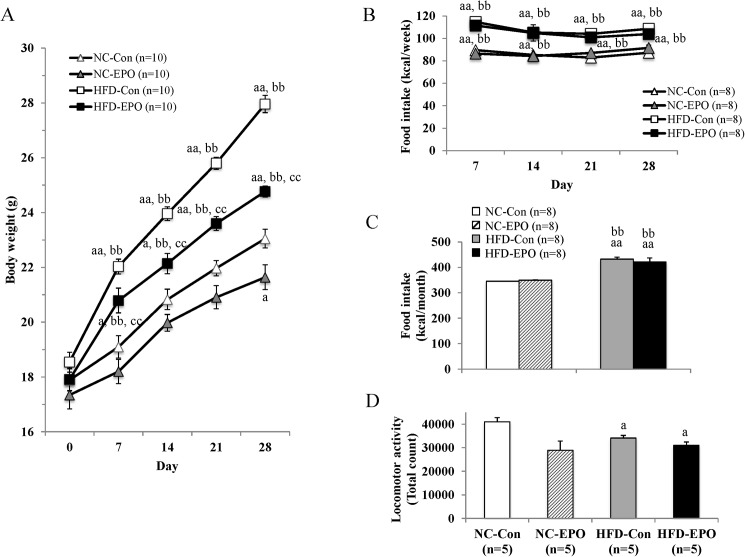
Effect of erythropoietin (EPO) on body weight gain. **(A)** Change in body weight. **(B)** Weekly food intake. **(C)** Total food intake. (**D)** Locomotor activity. Values are mean ± SE for 5–10 mice. ^a^*P* < 0.05 or ^aa^*P* < 0.01, vs. mice fed normal chow diet (NC-Con). ^b^P < 0.05 or ^bb^P < 0.01, vs. mice fed normal chow diet plus EPO (NC-EPO). ^c^P < 0.05 or ^cc^P < 0.01, vs. mice fed high-fat diet alone (HFD-Con).

**Fig 2:**
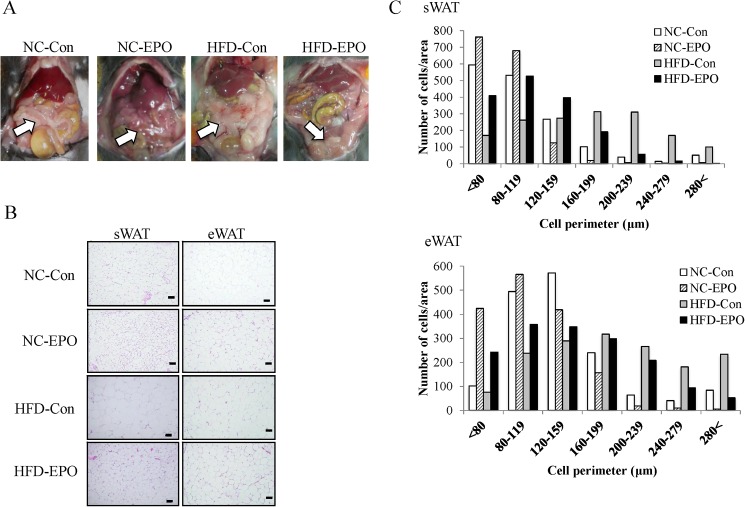
Effect of erythropoietin (EPO) on white adipose tissue (WAT). Histology of subcutaneous WAT (sWAT) and epididymal WAT (eWAT) were examined by HE staining. **(A)** Macroscopic images of eWAT in mice fed normal chow diet (NC-Con), mice fed high-fat diet alone (HFD-Con), and mice high-fat diet plus EPO (HFD-EPO). White arrow indicates eWAT. **(B)** Representative histology of NC-Con, HFD-Con and HFD-EPO mice in sWAT and eWAT. Scale bar = 50 μm. **(C)** The distribution of adipocyte perimeters in sWAT and eWAT were analyzed for each group.

The body weight of mice fed a high-fat diet (HFD) (referred to hereafter as HFD-Con mice) was significantly greater at the end of the experimental period than that of mice fed a normal chow (NC) (referred to hereafter as NC-Con mice). The body weight of mice fed HFD and injected with erythropoietin (EPO) (HFD-EPO mice) was significantly less than that of HFD-Con mice, even though there was no difference in caloric intake or locomotor activity between the two groups. The mice fed normal chow with EPO treatment (NC-EPO mice) showed a slightly, but significantly, lower body weight than that of NC-Con mice, despite there being no difference in food calorie intake between the two groups (Table 1, Fig 1). Epididymal WAT (eWAT) of HFD-EPO mice appeared to be smaller than that of HFD-mice (Fig 2A). The mass of eWAT and subcutaneous WAT (sWAT) in HFD-EPO mice was lower than that in HFD-Con mice (Table 1). Histological examination revealed that mean diameter of both subcutaneous and epididymal white adipocytes in HFD-Con mice was larger than that of both adipocytes in NC-Con and NC-EPO mice. The distribution patterns of the larger cells toward a smaller size in both of subcutaneous and epididymal white adipocyte was also evident in EPO-treated mice under a high fat diet (Fig 2B and 2C).

**Table 1:** Metabolic parameters of 8-week-old mice.

	n	NC-Con	NC-EPO	HFD-Con	HFD-EPO
Body weight (g)	10	23 ± 0.3	21.6 ± 0.5 ^a^	28 ± 0.3 ^aa^^,^ ^bb^	24.8 ± 0.2 ^aa^^,^ ^bb^^,^ ^cc^
Interscapular BAT mass (g)	9–14	0.06 ± 0.002	0.11 ± 0.007 ^aa^^,^ ^cc^	0.07 ± 0.003	0.13 ± 0.006 ^aa^^,^ ^cc^
Subcutaneous WAT mass (g)	5	0.21 ± 0.014	0.22 ± 0.023	0.89 ± 0.067 ^aa^^,^ ^bb^	0.7 ± 0.051 ^aa^^,^ ^bb^^,^ ^c^
Epididymal WAT mass (g)	9–15	0.24 ± 0.03	0.23 ± 0.024	0.85 ± 0.04 ^aa^^,^ ^bb^	0.61 ± 0.054 ^aa^^,^ ^bb^^,^ ^cc^
Glucose (mg/dl)	7–9	75.4 ± 9.5	51.6 ± 9.6	90.8 ± 8.9 ^b^	61.2 ± 6.9
Insulin (μU/ml)	7–9	12.4 ± 2.1	9.1 ± 1.1	13.6 ± 1.6	10.6 ± 1.0
HOMA-IR	7–9	2.3 ± 0.46	1.1 ± 0.23	3.1 ± 0.59 ^b^	1.5 ± 0.18 ^c^
Triglyceride (mg/dl)	4–8	52.3 ± 12.7	62 ± 18.1	37.8 ± 7.1 ^a^^,^ ^b^	45 ± 8.8 ^a^
Total cholesterol (mg/dl)	4–8	83.5 ± 2.3	86.8 ± 2.9	146.3 ± 4.9 ^aa^^,^ ^bb^	125.5 ± 3.8 ^aa^^,^ ^bb^^,^ ^cc^

Values are mean ± SE.

^a^*P* < 0.05 or ^aa^*P* < 0.01, vs. mice fed normal chow diet (NC-Con).

^b^P < 0.05 or ^bb^P < 0.01, vs. mice fed normal chow diet plus EPO (NC-EPO).

^c^P < 0.05 or ^cc^P < 0.01, vs. mice fed high-fat diet alone (HFD-Con).

**Fig 3:**
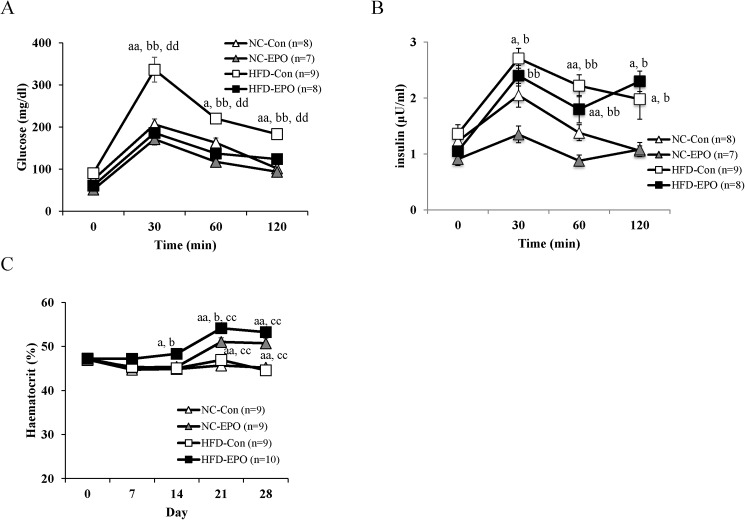
Effect of erythropoietin (EPO) on glucose tolerance and hematocrit levels (Ht). Four-week-old mice were treated with saline or with EPO (200 U/kg) for four weeks. Intra-peritoneal glucose tolerance tests (IPGTT) (1 g/kg) were performed after overnight fasting. (**A)** and **(B)** show blood glucose levels and insulin levels, respectively. (**C)** shows weekly Ht. Values shown are mean ± SE for 7–10 mice. ^a^*P* < 0.05 or ^aa^*P* < 0.01, vs. mice fed normal chow diet (NC-Con). ^b^P < 0.05 or ^bb^P < 0.01, vs. mice fed normal chow diet plus EPO (NC-EPO). ^c^P < 0.05 or ^cc^P < 0.01, vs. mice fed high-fat diet alone (HFD-Con). ^d^P < 0.05 or ^dd^P < 0.01, vs. mice fed high-fat diet plus EPO (HFD-EPO).

Blood glucose levels tended to be lower in HFD-EPO mice than in HFD-Con mice. Plasma insulin level was not significantly different among the four groups. HOMA-IR index, an indicator of insulin resistance, was significantly lower in HFD-EPO mice than in HFD-Con mice (Table 1). An intraperitoneal glucose tolerance test (IPGTT) to evaluate glucose tolerance and insulin sensitivity revealed that the blood glucose levels in HFD-EPO mice were notably lower than those of the HFD-Con mice following the glucose challenge, and the blood glucose levels in HFD-EPO mice were similar to those in NC-Con and NC-EPO mice (Fig 3A). During the IPGTT, change in plasma insulin levels did not differ between HFD-Con and HFD-EPO mice (Fig 3B).

Plasma TG levels did not show a difference between HFD-Con and HFD-EPO mice. Plasma T-Cho levels were significantly higher in HFD-Con mice than in NC-Con mice, but were significantly lower in EPO-treated groups (Table 1). EPO increased hematocrit values in both the group fed the normal chow and the high-fat diet (Fig 3C).

### EPO increased interscapular mass and temperature of brown adipose tissue (BAT), and facilitated energy expenditure by upregulating PRDM16, a key regulator of brown adipocyte differentiation in high-fat diet induced obese mice

**Fig 4:**
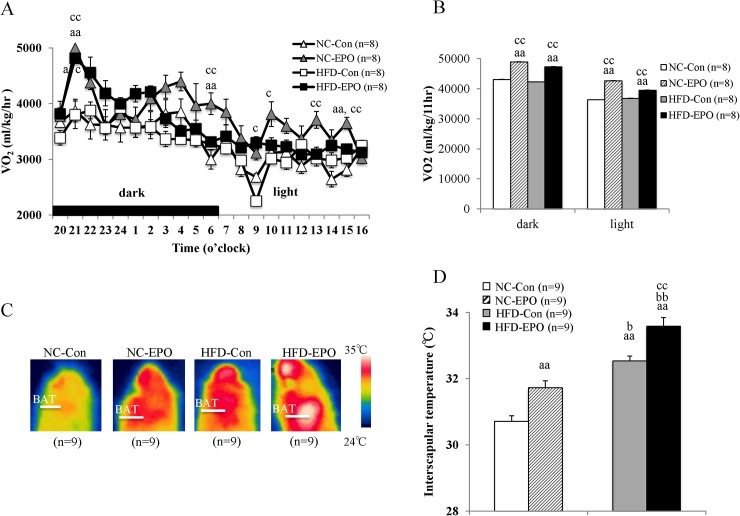
Effect of erythropoietin (EPO) on oxygen consumption and interscapular BAT. **(A)** 22-h Oxygen consumption. **(B)** Oxygen consumption in dark and light phases. **(C)** Representative infrared thermal images of normal chow diet mice (NC-Con), EPO treated normal chow diet mice (NC-EPO), high-fat diet mice (HFD-Con) and EPO-treated high-fat diet mice (HFD-EPO). **(D)** Interscapular surface temperature. Values shown are mean ± SE for 8–9 mice. ^a^*P* < 0.05 or ^aa^*P* < 0.01, vs. NC-Con. ^b^P < 0.05 or ^bb^P < 0.01, vs. NC-EPO. ^c^P < 0.05 or ^cc^P < 0.01, vs. HFD-Con.

**Fig 5:**
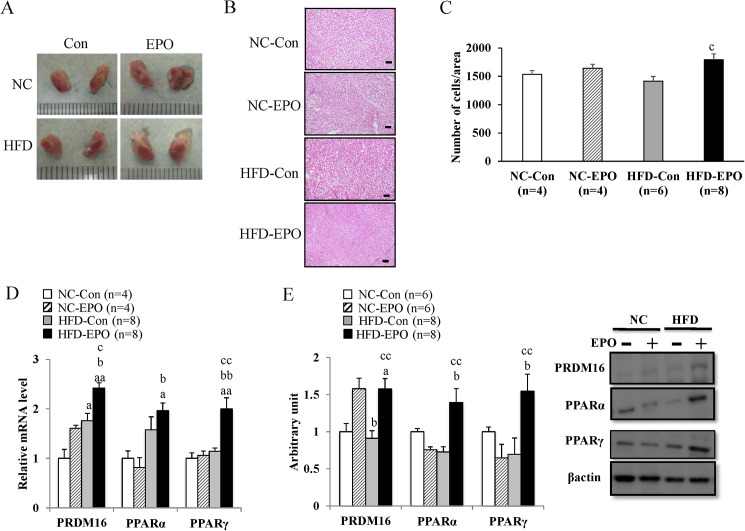
Effect of erythropoietin (EPO) on differentiation related genes in interscapular BAT (iBAT). Histology of iBAT was examined by HE staining (Scale bar = 50 μm). **(A)** Macroscopic images of iBAT. **(B)** Representative histology of iBAT in normal chow diet mice (NC-Con), high-fat diet mice (HFD-Con), and EPO-treated high-fat diet mice (HFD-EPO). **(C)** The number of cells/area in iBAT was counted. **(D)** Real-time PCR experiments. **(E)** Western blot analysis. Values are mean ± SE for 4–8 mice. ^a^*P* < 0.05 or ^aa^*P* < 0.01, vs. NC-Con. ^b^P < 0.05 or ^bb^P < 0.01, vs. mice fed normal chow diet plus EPO (NC-EPO). ^c^P < 0.05 or ^cc^P < 0.01, vs. mice HFD-Con.

In both the dark and light phases, oxygen consumption (VO_2_), an indirect measure of metabolism, was significantly higher in EPO-treated mice than in EPO-untreated mice fed both NC and HFD (Fig 4A and 4B). Surface temperature of the regions around interscapular brown adipose tissue (iBAT) in EPO-treated mice was significantly higher than that in untreated mice (Fig 4C and 4D). Further, iBAT mass of EPO-treated mice appeared to be larger than in untreated mice (Fig 5A). The weight of iBAT in HFD-EPO mice was significantly higher than that in HFD-Con mice, and the weight of iBAT in NC-EPO mice was also significantly higher than that of NC-Con mice (Table 1). There was a slight but significant decrease in the amount of lipid accumulation in BAT cells in HFD-EPO mice as compared to HFD-Con mice (Fig 5B and 5C).

Based on the result of enlargement of iBAT mass in EPO treated mice, we hypothesized that EPO exerted a positive regulatory effect on brown adipocyte differentiation. We evaluated gene and protein expressions of transcriptional factor PRDM16, a master regulator of brown adipocyte differentiation [33]. The mRNA expression of PRDM16 in HFD-EPO mice was 1.3-fold higher than that of HFD-Con mice (p < 0.05, Fig 5D). The protein levels of PRDM16 in HFD-EPO mice were 1.7-fold higher than those in HFD-Con mice (p < 0.01, Fig 5E). The peroxisome proliferator-activated receptors (PPARs) are a group of nuclear receptor proteins and are associated with transcription regulation, and PPARα and PPARγ are involved in brown adipocyte differentiation in concert with PRDM16 [33–35]. The mRNA expression of PPARγ was significantly higher in HFD-EPO mice than in HFD-Con mice (Fig 5D). The protein expression of PPARα and PPARγ in HFD-EPO mice were found to be significantly higher than in HFD-Con mice (Fig 5E).

**Fig 6:**
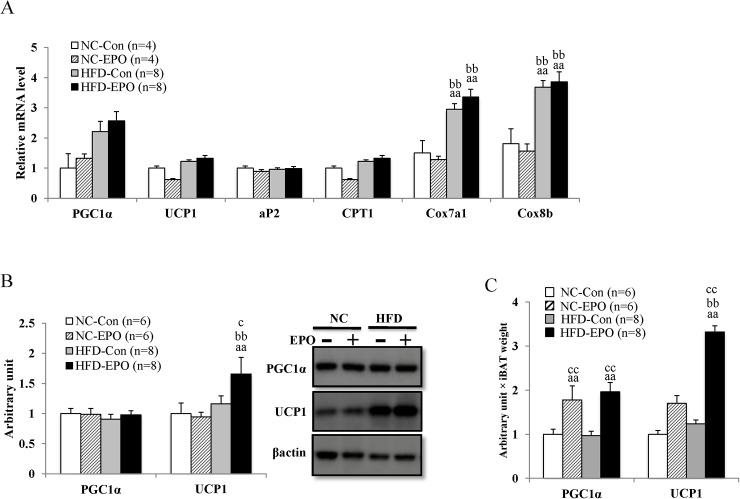
Effect of erythropoietin (EPO) on the expressions of PGC1α and UCP1 in interscapular BAT (iBAT). **(A)** Real-time PCR experiments. **(B)** Western blot analysis. **(C)** Arbitrary unit multiplied by iBAT weight. Values shown are mean ± SE for 4–8 mice. ^a^*P* <0 .05 or ^aa^*P* < 0.01, vs, mice fed normal chow diet (NC-Con). ^b^P < 0.05 or ^bb^P < 0.01, vs. mice fed normal chow diet plus EPO (NC-EPO). ^c^P < 0.05 or ^cc^P < 0.01, vs. mice fed a high-fat diet alone (HFD-Con).

BAT thermogenesis function is mediated by UCP1, which is uniquely present in inner membrane of mitochondria in brown adipocytes. UCP1 uncouples mitochondrial proton transport from ATP synthesis, thereby dissipating energy as heat [31,36]. PGC1α functions as a transcriptional coactivator that is needed for expression of the UCP1 gene [37]. Here, mRNA and protein expression of PGC1α was not significantly different among the four groups, nor was the mRNA level of UCP1 (Fig 6A). However, the protein expression of UCP1 was slightly but significantly higher in HFD-EPO mice than HFD-Con mice (Fig 6B). When we calculated the total amount of PGC1α and UCP1 proteins while taking the mass sizes into account, the PGC1α and UCP1 proteins in HFD-EPO mice were much higher than those of HFD-Con mice (Fig 6C).

Adipocyte protein 2 (aP2), an intracellular lipid binding protein, binds fatty acids and transports them to various compartments within an adipocyte [38]. The mRNA level of aP2 was not significantly different among iBAT from the four groups. Carnitine palmitoyltransferase I (CPT1) is the rate-limiting enzyme for fatty acid β- oxidation. The mRNA level of CPT1 was not different in iBAT from HFD-Con and HFD-EPO mice. The cytochrome c oxidase (Cox) is the terminal component of the mitochondrial respiratory chain. The mRNA levels of Cox subunit 7a1 and 8b were not significantly different between HFD-Con mice and HFD-EPO mice in iBAT (Fig 6A).

### EPO upregulates PRDM16 via erythropoietin receptor (EpoR)/ STAT3 pathway of iBAT in high-fat diet-induced obese mice

**Fig 7:**
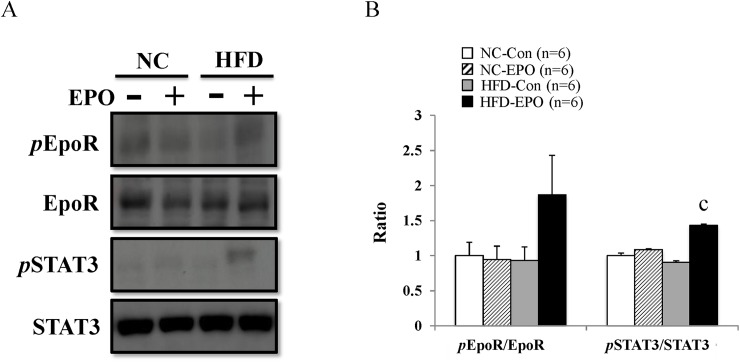
Effect of erythropoietin (EPO) on EpoR/STAT3 axis in interscapular BAT. **(A)** Western blot analysis. **(B)** *p*EpoR/EpoR and *p*STAT3/STAT3 ratios were calculated. Values given are mean ± SE for 6 mice. ^a^*P* < 0.05 or ^aa^*P* < 0.01, vs. mice fed normal chow diet (NC-Con). ^b^P < 0.05 or ^bb^P < 0.01, vs. mice fed normal chow diet plus EPO (NC-EPO). ^c^P < 0.05 or ^cc^P < 0.01, vs. mice fed high-fat diet alone (HFD-Con).

EPO tended to increase *p*EpoR/EpoR ratio under a high-fat diet (Fig 7A and 7B). EpoR activates JAK2 tyrosine kinase, which activates several different intracellular pathways, with STATs occurring downstream of one of these. JAK2 phosphorylates STATs, leading to dimerization. The STAT dimer is translocated to the nucleus and binds to the DNA to regulate transcription [39, 40]. A recent report has demonstrated that STAT3 enhances stability of PRDM16 in murine brown adipocyte [41]. In our study, EPO significantly increased *p*STAT3/STAT3 ratio under a high-fat diet (Fig 7A and 7B).

### EPO upregulates PRDM16 via β-adrenergic receptor/Mef2c/ miR-133 cascade of interscapular brown adipose tissue (iBAT) in high-fat diet induced obese mice

**Fig 8:**
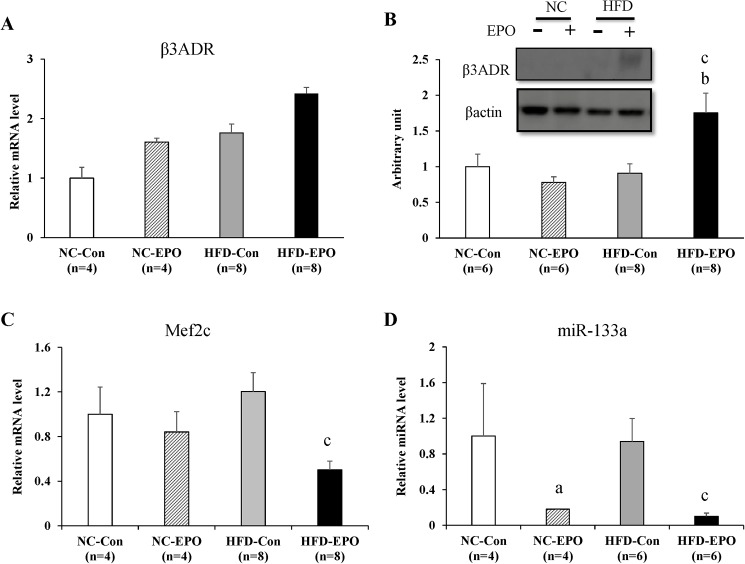
Effect of erythropoietin (EPO) on the β-adrenergic receptor/Mef2/miR-133 pathway in interscapular BAT. (A) Real-time PCR experiments. (B) Western blot analysis. (C) Real-time PCR experiments. (D) microRNA analysis experiments. Values given are mean ± SE for 4–8 mice. ^a^*P* < 0.05 or ^aa^*P* < 0.01, vs. mice fed normal chow diet (NC-Con). ^b^P < 0.05 or ^bb^P < 0.01, vs. mice fed normal chow diet plus EPO (NC-EPO). ^c^P < 0.05 or ^cc^P < 0.01, vs. mice fed high-fat diet alone (HFD-Con).

Previous reports have demonstrated that β-adrenergic stimulation suppresses microRNA-133 (miR-133) expression in a myocyte enhancer factor 2 (Mef2c)-dependent manner, which results in direct de-repression of PRDM16 expression in brown adipose tissue (BAT) [42–45]. β3-adrenergic receptor (β3ADR) mRNA expression was not different between HFD-Con and HFD-EPO mice (Fig 8A). However, protein level of β3ADR in HFD-EPO mice was significantly higher than that of HFD-Con mice (Fig 8B). Mef2c mRNA expression in HFD-EPO mice was significantly lower than in HFD-Con mice (Fig 8C). The level of miR-133a was markedly decreased by EPO under both normal chow and high-fat diet conditions (Fig 8D).

**Fig 9:**
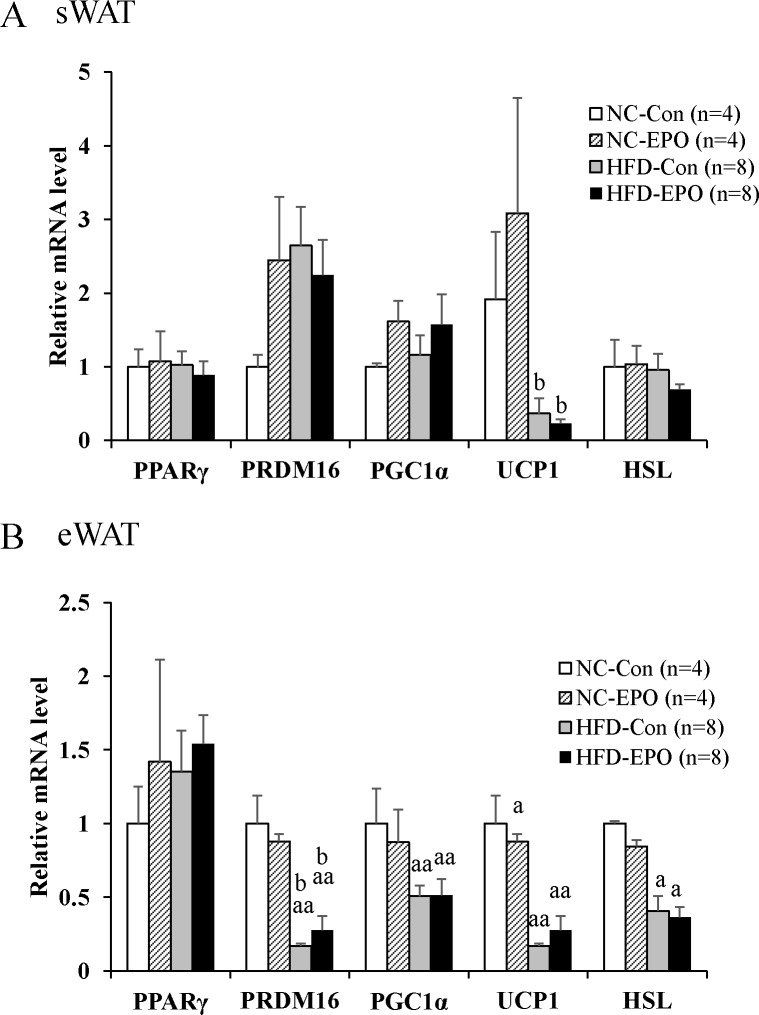
Effect of erythropoietin (EPO) on differentiation-, lipolysis- and thermogenesis-related genes in white adipose tissue (WAT). **(A)** Real-time PCR experiments in subcutaneous WAT (sWAT). **(B)** Real-time PCR experiments in epididymal WAT (eWAT). Values given are mean ± SE for 4–8 mice. ^a^*P* < 0.05 or ^aa^*P* < 0.01, vs. mice fed normal chow diet (NC-Con). ^b^P < 0.05 or ^bb^P < 0.01, vs. mice fed normal chow diet plus EPO (NC-EPO). ^c^P < 0.05 or ^cc^P < 0.01, vs. mice fed high-fat diet alone (HFD-Con).

We examined whether EPO induces the appearance of beige adipocyte in WAT (i.e. browning of WAT). We did not find that EPO had any effect on any BAT-specific gene (PRDM16, PGC1α, or UCP1) of HFD-Con mice. We also evaluated the influence of EPO on adipogenesis and lipolysis of WAT, but found that EPO had no effect on a key regulator of adipocyte development PPARγ or lipolytic enzyme HSL (Fig 9A and 9B).

### EPO promoted secretion of iBAT-derived fibroblast growth factor 21 (FGF21) of HFD-Con mice

**Fig 10:**
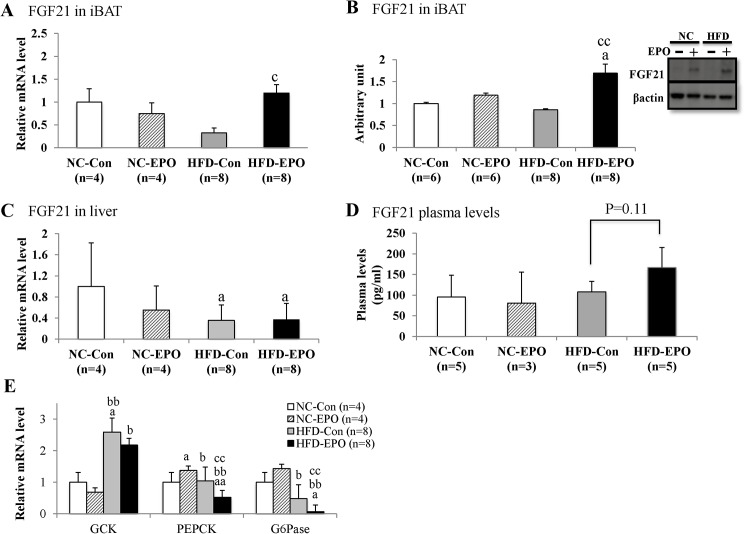
Effect of erythropoietin (EPO) on expression/secretion of FGF21 in interscapular BAT (iBAT) and the liver and gluconeogenesis-related genes in the liver. **(A)** Real-time PCR experiments in iBAT. **(B)** Western blot analysis. (**C)** Real-time PCR experiments in liver tissue. (**D)** Plasma levels of FGF21. **(E)** Real-time PCR experiments in liver tissue. Values given are mean ± SE for 3–8 mice. ^a^*P* < 0.05 or ^aa^*P* < 0.01, vs. mice fed normal chow diet (NC-Con). ^b^P < 0.05 or ^bb^P < 0.01, vs. mice fed normal chow diet plus EPO (NC-EPO). ^c^P < 0.05 or ^cc^P < 0.01, vs. mice fed a high-fat diet alone (HFD-Con).

FGF21 is known to be a metabolic regulator in the control of glucose homeostasis and insulin sensitivity [43], and the liver is considered to be the main site of production and release of FGF21 into circulation. Evidence that BAT also releases FGF21 has gradually accumulated [23–25]. FGF21 mRNA and protein expression in iBAT of HFD-EPO mice were both significantly higher than those of HFD-Con mice, whereas FGF21 mRNA expression in the liver was not different between HFD-Con mice and HFD-EPO mice (Fig 10A–10C). Plasma FGF21 levels in HFD-EPO mice tended to be higher than in HFD-Con mice (p = 0.11, Fig 10D).

FGF21 increases hepatic insulin sensitivity, resulting in a reduction of gluconeogenesis in the liver [27, 28]. We examined gluconeogenesis-related genes in the liver. Glucokinase (GCK) catalyzes the initial step of glycolytic pathway, facilitating phosphorylation of glucose to glucose 6-phosphate. Phosphoenolpyruvate carboxykinase (PEPCK) and glucose-6-phosohatase (G6pase) catalyze the first and last steps of the gluconeogenesis, respectively [44]. Here, there was no difference in GCK mRNA expression between HFD-Con and HFD-EPO mice. However, mRNA expression of PEPCK and G6Pase in HFD-EPO mice was significantly lower than that in HFD-Con mice (Fig 10E).

## Discussion

Erythropoietin treatment (EPO) reduced body weight and HOMA-IR (index of insulin resistance), and reduced white fat accumulation in high-fat diet (HFD)-induced obese mice in our study (Table 1, Figs 1A and 2A). Although our dosage was lower than in past investigations, the anti-obesity effects exerted by EPO in our study were consistent with previous studies in animal models [2,3,10–12,14]. Furthermore, we showed that EPO reduced blood glucose levels during an intraperitoneal glucose tolerance test (IPGTT) without changing serum insulin levels in obese mice (Fig 3A and 3B). This effect of EPO in improving glucose tolerance is also consistent with previous studies [2,3,8,10,11,14]. Here, we provide new evidence that classical brown adipose tissue (BAT) plays an important role in how EPO alleviates obesity and glucose homeostasis in mice.

Based on our findings that EPO did not influence locomotor activity or food intake under HFD feeding (Fig 1B–1D), we posit that facilitation of energy expenditure can be attributed to reducing the weight gained by HFD-fed mice. We further suggest that BAT is involved in the EPO-mediated anti-obesity effect observed in our study, as BAT increases energy expenditure in organisms by dissipating chemical energy as heat (i.e. thermogenesis). BAT has been long believed to disappear after infancy; however, recent studies using positron emission tomography/computed tomography (PET/CT) have demonstrated that adult humans still possess metabolically active BAT [17–19]. Since obesity stems from an imbalance between energy intake and expenditure, activation of BAT has received considerable attention as an obesity treatment [20,22, 46]. Thermogenic adipocytes existing in rodents and humans is divided into the two categories of classical brown adipocyte and beige (also referred as brite) adipocytes. Classical brown adipocytes arise from myogenic factor 5 (Myf5)-positive progenitors cells during fetal development, and are found mainly in the interscapular region of rodents and infant humans [21,22].

We demonstrated that EPO increased oxygen consumption accompanied with increased interscapular BAT (iBAT, or the main part of classical BAT), temperature, and mass (Table 1, Figs 4 and 5A). These findings suggest that iBAT is associated with EPO’s facilitation of energy expenditure, and motivated us to investigate the molecular mechanism by which EPO induced the phenomenon. We showed that PRD1-BF1-RIZ1 homologous domain containing 16 (PRDM16), which drives the differentiation of myogenic fat precursors to brown adipocytes [33], is significantly upregulated in HFD-EPO mice as compared with that in HFD-Con mice (Fig 5D and 5E). We next investigated which signal transduction pathway might trigger the upregulation of PRDM16.

Previous research has documented that erythropoietin receptor (EpoR) is located in skeletal muscle, white adipose tissue, brain, liver, and heart as well as bone marrow [3,11]. The distribution of EpoR in multiple tissues contributes to pleiotropic physiological effects on the body beyond the hematopoietic action [47, 48]. Although it has not been established that EpoR exists in classical BAT, EpoR protein was detected in iBAT in our study. Furthermore, we demonstrated that protein expression of *p*EpoR, the initial regulator of EpoR/JAK2/STAT3 signaling, tended to be higher in HFD-EPO mice than in HFD-Con mice (Fig 7). The JAK-STAT signaling pathway is evolutionarily conserved in eukaryotes and is associated with cell growth, survival, differentiation, and development [39, 40]. A recent report has demonstrated that STAT3 binds and stabilizes PRDM16 in cultured brown adipocytes from iBAT of mice [41]. In our study, the *p*STAT3/STAT3 ratio was significantly increased by EPO-treatment under a high-fat diet (Fig 7).

β-adrenergic stimulation after cold exposure is reported to suppress myocyte enhancer factor 2 (Mef2) expression, which results in remarkable downregulation of microRNA-133 (miR-133) in BAT [42, 44]. The downregulation of mir-133 directly de-repression of PRDM16 expression [43,45]. In our study, EPO significantly increased protein expression of β3-adrenergic receptor (β3ADR) under a high-fat diet (Fig 8B). Furthermore, the expression of both Mef2c mRNA and miR-133a was significantly decreased in EPO-treated mice under a high-fat diet (Fig 8C and 8D).

These data suggest that EPO upregulates PRDM16 through EpoR/STAT3 and β-adrenergic receptor/Mef2/miR-133 signaling pathway, which results in the enlargement of iBAT mass. In our study, the expression of uncoupling protein1 (UCP1) protein, essential for thermogenesis in BAT [36], was slightly but significantly higher in HFD-EPO mice than in HFD-Con mice (Fig 6B). However, when we took the different mass sizes into account, the total amount of UCP1 protein was much higher in HFD-EPO mice than in HFD-Con mice (Fig 6C). Therefore, the quantitative upregulation of UCP1 is attributable largely to increased thermogenesis in our study. Although one previous paper reported EPO induced WAT browning in mice [11], the phenomenon was not observed in our experiment (Fig 9).

The present study showed that weight of subcutaneous and epididymal WAT is significantly lowered by EPO treatment in high-fat diet-induced obese mice (Table 1). Moreover, histological analysis revealed that EPO treatment reduced the mean size of white adipocytes (Fig 2B and 2C). EPO effects on the differentiation of WAT have remained controversial. Teng et al. have reported that EPO treatment downregulated PPARγ expression, which is a master regulator of white adipocyte differentiation and reduced white adipocyte differentiation in 3T3-L1 cells [3]. Meanwhile, Christensen et al. have demonstrated that EPO had no effect on p38 α mitogen-activated protein kinase (p38αMAPK), which is downstream of EpoR and regulates PPARγ expression [49] in human WAT [15]. Our data showed that EPO did not have an effect on PPARγ gene expression in mice WAT (Fig 9). Furthermore, Christensen et al. have demonstrated that EPO did not affect hormone sensitive lipase (HSL), which is essential for lipolysis in human WAT [15]. Consistent with this finding, the expression of HSL in WAT is not different between the EPO-untreated groups and the EPO-treated groups in our study (Fig 9). We assumed that the reduction of WAT observed in EPO-treated mice under HFD was due to facilitation of energy expenditure, and not due to lipolysis or suppression of white adipogenesis.

FGF21, which is an endocrine member of FGF family, is involved in improvement of insulin resistance and glucose metabolism. Liver tissue is known to be main site of FGF21 production; however, recent reports have demonstrated that BAT also produces FGF21 [23, 24]. β3-adrenergic stimulation is known to induce FGF21 expression of BAT [25], and the finding that EPO increased β3ADR expression in our study (Fig 8B) motivated us to examine whether EPO influences FGF21 expression in BAT. In our study, under a high fat diet EPO significantly increased mRNA and protein expressions of FGF21 in iBAT (Fig 10A and 10B), but it did not change in the liver (Fig 10C). Plasma levels of FGF21 tended to be higher in EPO-treated mice fed a high-fat diet (Fig 10D), suggesting that EPO activated the production and excretion of FGF21 of iBAT in our study. We also showed that EPO suppressed mRNA expression of PEPCK and G6pase in the liver (presumably leading to a reduction in liver gluconeogenesis) under a high-fat diet (Fig 10E). Since the FGF21’s improvement of insulin resistance is largely attributed to enhanced insulin action in the liver [26–30], we suggest that the EPO’s enhancement of BAT derived-FGF21 secretion contributes to the reduction of gluconeogenesis of liver in induced obese mice on a high-fat diet.

We did not examine the effect EPO has on brown adipocyte in vitro, which would greatly contribute to elucidating whether EPO acts on iBAT directly or indirectly. Future experiments with knockdown models for EpoR and β3ADR would support our findings that EPO acts on iBAT through these receptors. In addition, how EPO eminently exerts anti-obesity and anti-diabetic effect only in high-fat diet conditions should be addressed in future studies.

Administration of EPO inevitably induces elevation of hematocrit levels (Ht), which can cause undesirable side effects such as arterial hypertension and thromboembolism. Since the dosage of EPO in our study was very close to the current clinical dosage in neonate anemia, and did not induce severe elevation of Ht (Fig 3C), we believe that the dosage of EPO used here might also safely be used clinically in obese or diabetic children. Meanwhile, for adults who have underlying diseases such as vascular disease, anti-obesity and anti-diabetic drug treatments which act on non-haemopoietic systems are necessary. One clinical study has demonstrated that a non-erythropoietic peptide engineered from EPO could have beneficial effects on metabolic parameters and neuropathic symptoms in adults with Type 2 diabetes [50]. However, no clinical studies have yet examined EPO’s anti-obesity effect in humans. Our study has provided a strong basis for further investigation of the anti-obesity and diabetic effects of EPO, and we hope that this will contribute to the development of new safe and effective drugs against obesity and diabetes.

In summary, we found that: 1) EPO facilitates energy expenditure by increasing classical BAT mass; 2) EPO stimulates EpoR/STAT3 and β-adrenergic receptor/Mef2c/miR-133 pathways, resulting in enhancement of PRDM16 of classical BAT; 3) EPO promoted secretion of classical BAT’s derived-FGF21; and 4) EPO ameliorated obesity and glucose homeostasis in high-fat diet-induced obese mice. These findings suggest that EPO has potential to increase classical BAT capacity and promote endocrine function to protect against obesity and diabetes.

## Supporting information

S1 Table Sequences of primers for quantitative RT-PCR. (PDF)
