# Supplementary material for: Erythropoietin (EPO) ameliorates obesity and glucose homeostasis by promoting thermogenesis and endocrine function of classical brown adipose tissue (BAT) in diet-induced obese mice
Source: PLoS One. 2017 Mar 13;12(3):e0173661. doi: 10.1371/journal.pone.0173661 (PMC5348037; doi:10.1371/journal.pone.0173661)
Supplement: S1 Table — (PDF) [file pone.0173661.s001.pdf]

Primers	Sequnces
PRDM16 forward	CAGCACGGTGAAGCCATTC
PRDM16 reverse	GGCGTGCATCCGCTTGT
PPARα forward	ATGCCAGTACTGCCGTTTTC
PPARα reverse	CCGAATCTTTCAGGTCGTGT
PPARγ forward	CCAGAGTCTGCTGATCTGC
PPARγ reverse	GCCACCTCTTTGCTCTGCTC
PGC1α forward	TGAACGCACCTTAAGTGT GGA
PGC1α reverse	GGGTTATCTTGGTTGGCTTTATGA
UCP1 forward	AGGCTTCCAGTACCATTAGGT
UCP1 reverse	CTGAGTGAGGCAAAGCTGATTT
aP2 forward	CAGAAGTGGGATGGAAAGTCG
aP2 reverse	CGACTGACTATTGTAGTGTTTGA
CPT1 forward	CCACTGATGAAGGAGGGAGAC
CPT1 reverse	TATGGGTTGGGGTGATGTAGA
Cox7a1 forward	CCGACAATGACCTCCCAGTA
Cox7a1 reverse	TGTTTGTCCAAGTCCTCCAA
Cox8b forward	GAACCATGAAGCCAACGACT
Cox8b reverse	GCGAAGTTCACAGTGGTTCC
β3ADR forward	TCTAGTTCCCAGCGGAGTTTTCATCG
β3ADR reverse	CGCGCACCTTCATAGCCATCAAACC
Mef2c forward	TCTGCCCTCAGTCAGTTGG
Mef2c reverse	CGTGGTGTGTTGTGGGTATC
FGF21 forward	AGATCAGGGAGGATGGAACA
FGF21 reverse	TCAAAGTGAGGCGATCCATA
GCK forward	CAACTGGACCAAGGGCTTCAA
GCK reverse	TGTGGCCACCGTGTCATTC
G6Pase forward	TCGTGGCTGGAGTCTTGTCA
G6Pase reverse	GGCTGGCAAAGGGTGTAGTG
PEPCK forward	AGCCTTTGGTCAACAACTGG
PEPCK reverse	TGCCTTCGGGGTTAGTTATG
β-actin forward	GGCTGTATTCCCCTCCATCG
β-actin reverse	CCAGTTGGTAACAATGCCATGT

Supplementary Table S1. Sequences of primers for quantitative RT-PCR
